# "The Hospital" Nursing Mirror

**Published:** 1899-11-25

**Authors:** 


### "The Hospital" Nursing Mirror.

Being the Nursing Section of "The Hospital."

[Contributions for this Section of "The Hospital" should be addressed to the Editor, The Hospital, 28 & 29, Southampton Street, Strand, London, W.C., and should have the word "Nursing" plainly written in left-hand top corner of the envelope.]

### Notes on News from the Nursing World.

### THE PRINCESS OF WALES AND THE WAR NURSES.

Although the message of the Princess of Wales which Colonel Young, of the Red Cross Society, read at a meeting at Cape Town on behalf of the sick and wounded, "I wish God-speed to you in your efforts to allay suffering, and I trust that your work will be crowned with complete success," was to the people of Cape Colony in general, it was to the nurses in particular. The same may be said of the sympathetic and characteristic letter from Miss Florence Nightingale to the following effect: "This is a sad and painful business, but how much good it has called forth! May we hope that nurses and everybody will prove themselves worthy of the great opportunity afforded by God's goodness. I wish I could go, but am chained to my room with illness. Three cheers for you wherever you go—cheers to strengthen and not disturb the sick." This week the "Princess of Wales" vessel starts for South Africa. She is fitted with 200 beds, and will bring from Cape Town to England those of the sick and wounded who are able to undertake the journey. The four nurses on board have each a cabin, with a small tea-room in the port alley way. The ship has been painted entirely white, with the red cross in various parts.

### A PLETHORA OF NURSES.

The Central Red Cross Society have been inundated with applications from persons wanting to go out as nurses to the seat of war. It is stated that if they had chosen they might "have engaged a nurse for practically every individual soldier likely to be wounded in the campaign." As may be imagined, some of the applications have been from individuals without the slightest qualifications for the work. Mention is made, for example, of a lady and her daughter who, in offering to go out, said that "they had never done any nursing before, but were thoroughly domesticated." When will ladies of this class understand that nursing is a serious profession, and that it needs training and experience as much as any other to practise it successfully?

### THE AMERICAN HOSPITAL SHIP.

The splendid sum of £1,600 was realised by the Café Chantant on Saturday in aid of the American hospital ship. Moreover, people got a good deal of pleasure out of their expenditure, for the event was quite a big society affair; and the Prince of Wales, the Princess Christian, and the Duke of Cambridge were among the representative company. Unfortunately, Lady Randolph Churchill was too concerned about the fate of her gallant son to be present. Happily, it has since been ascertained that Mr. Winston Churchill, though captured by the Boers after his courageous achievement, is not wounded. As already intimated, the matron on the "Maine," Miss Hibbard, is one of the most distinguished nurses in the United States, and we understand that all the staff have been selected specially on account of the proficiency they displayed during the late Spanish-American War.

### THE BOER GOVERNMENT AND BRITISH NURSES.

The skilled and complete staff of British nurses at Johannesburg Hospital has been replaced by a number of Boer ladies whose knowledge of nursing is apparently limited to attendance at a course of ambulance lectures. This is the most serious point of the account given in another column of the eviction under the auspices of an agent of President Kruger, though the whole of it will be read with the utmost indignation. The pity is that the sufferers from the astounding action of the Boer authorities will be persons who had no part nor lot in it—chiefly, we may presume, wounded Boers who are likely to find the ministrations of Dutch ladies, who have listened to a few lectures, very different from those of trained nurses willing and able to afford them all possible attention and relief.

### NURSING THE WOUNDED AT LADYSMITH.

Striking confirmation of the statement that the wounded Boers are as well cared for as the wounded English, is given in a letter from one of the nursing sisters at the Military Hospital, Ladysmith, to Sir John Voce Moore, the late Lord Mayor of London. Writing under date of October 26th, after the battle of Elands Laagte, she says:—

"All night on Saturday the wounded streamed in, and all day on Sunday we not only had our beds all full, but they were lying about the floor as thickly as we could put them. We could only just step between each to administer to their wants until their wounds could be dressed. Wounded, wet, and cold, some had been lying thirty hours on the wet ground. They told a sad tale of suffering, but without a murmur of complaint. Their bravery and endurance are marvellous. Some were so terribly wounded that they succumbed a few hours after their wounds were dressed. . . . We have several wounded Boer patients, and it is really amusing to see our large-hearted Tommy Atkinses fraternising with the enemy. A touching little scene happened yesterday. One of the Gordons had his arm amputated. A Boer in the next bed had had his arm taken off in exactly the same place. I took charge of the latter as he was brought down from the theatre, and on his becoming conscious the two poor fellows eyed each other very much, till our good-natured Tommy could bear it no longer. 'Sister,' he called, 'give him two cigarettes out of my box, and tell him I sent them. Here is a match; light one for him.' I took the cigarettes and the message to the Boer, and he turned and looked at Tommy in amazement, and then, quite overcome, he burst into tears, and Tommy did the same, and I am afraid I was on the point of joining in the chorus, but time would not permit it."

It is satisfactory to learn from the nursing sister that there is a splendid staff of skilled surgeons at the Ladysmith Military Hospital, and that the nursing staff is all that could be desired.

### THE PERSONNEL OF THE NURSES AT LADYSMITH.

The nursing staff at the Ladysmith Military Hospital consists of Superintendent Sister E. A. Dowse, Sister Bourchier, Nursing Sisters Noble and Band, and Sisters Ludlow, M. Charlstone, E. Borlase, Lees, and Hill.

Miss Dowse has been a superintendent of the Army Nursing Service since July, 1898. She was trained at St. Mary's Hospital, and was subsequently staff nurse from 1879 to 1885, when she became a sister of the Army Nursing Service. Sister Bourchier, formerly Miss Wallace, joined the service in 1881. Sister Noble, of the Army Nursing Service Reserve, was trained at the Royal Hospital, Sheffield, where she afterwards served as staff nurse for a year and charge nurse for a year. She was then charge nurse at the Hospital and Dispensary, Beverley, and at Fir Vale Infirmary, Sheffield, finally becoming matron of the Beverley Hospital in July, 1896, an appointment she relinquished in order to proceed to South Africa.

### THE COLONIAL OFFICE AND SOUTH AFRICA.

APPLICATIONS for particulars about nursing arrangements in South Africa having been made to the Colonial Office, Lord Ampthill, writing on behalf of the Colonial Society, asks us to make known the fact "that the Colonial Office is not in any way concerned in the nursing arrangements in connection with the troops in South Africa, and is unable to supply any information on the subject."

### THE QUESTION OF NUNS AND NURSING AT GALWAY.

WITH reference to the refusal of the Irish Local Government Board to sanction the appointment of Sister Mary Bernard Ryan as matron of Galway General Hospital, to which we recently alluded, we are informed that the hospital board consists of a number of guardians from each union in the county, Galway being only one out of ten. According to the *Freeman's Journal*, the Guardians of the Ballinasloe Union—another of the unions concerned—calls upon the Hospital Board to "insist upon" the appointment of Miss Ryan. The refusal of the Local Government Board is, however, the secretary states, due to the fact that the appointment does not fulfil the following conditions of the Galway Hospital Act, 1892:—

"Article 39.—No person shall be appointed by the board of management as nurse who shall not have undergone three years' training in or have obtained from some recognised public hospital a certificate of training (where such certificates are issued) showing that she is qualified to discharge the duties she will be called upon to perform in the hospital. Provided always that the board of management may appoint from time to time such uncertificated or untrained nurses or probationers as may be deemed necessary to act as assistants.

"Article 40.—No person shall be appointed as matron who shall not be a certificated or trained nurse within the meaning of Article 39."

As, according to Article 30, supreme power is vested in the Local Government Board, which can thus decline to give its consent to the appointment of any nurses of whom it does not approve, we do not see how it is possible for the Galway Board to "stand out" against its decision.

### MORE "HUMOROUS" POOR LAW GUARDIANS.

THE Colchester Guardians do not seem to have a much better sense of dignity than the West Ham Guardians, to whose "pleasantries" we called attention some little time ago. At a meeting the other day, at which it was decided to invite certain nurses who were candidates for a vacancy to attend before the Board, Mr. Cant observed "that if all three nurses came it would be a case of selecting the best looking one." At this brilliant sally, the report says, there was "laughter," whereupon Mr. Littlebury remarked, "You shouldn't take the face of a woman for her work," and there was "more laughter." It cannot be expected that self-respecting nurses will care to undergo the scrutiny of Guardians who refer to them much in the same manner as if they were cattle about to compete in the show-yard.

### THE NEW SOUTH WALES TRAINED NURSES' ASSOCIATION.

THE formation of the New South Wales Trained Nurses' Association has already been mentioned in these columns. We have now received a copy of the constitution of the organisation, which, however, was foreshadowed in THE HOSPITAL "Nursing Mirror" of September 2nd. Contrary to expectation, there has been no modification in the qualification for registration. Thus, according to Article 16, candidates "must produce proof that they have been engaged for three years in work in general hospitals recognised by the council, and containing at least 40 beds." Article 17 runs: "Any nurse who has been trained at a training school for a period of two years, and has obtained her certificate as a nurse from that training school, shall be eligible for registration up to January 1st, 1900," while a nurse trained in a private hospital, with not less than 10 beds, must have served five years and have a certificate from a matron who herself holds a certificate from a training school. The council, very properly, have power to remove from the register the name of any nurse who, after full inquiry, may prove herself to be unworthy of trust. Six matrons, the lady superintendent of a private hospital, two sisters, and three nurses attached to nurses' homes, are included on the council.

### NURSING IN SHREWSBURY.

ATTENTION has been called to the lack of provision for the nursing of the sick poor of Shrewsbury at their homes in cases of contagious diseases. There is a nursing association in the town which was established in 1879 "to furnish the services of trained nurses to visit and nurse the sick poor of Shrewsbury in their own homes." But the rules of the association will not permit them to attend cases of scarlet fever, small-pox, and typhus. A correspondent of a Shrewsbury paper mentions two instances of fever attacking several persons in the house, and in neither of these was it possible to obtain the service of a nurse. "The lack of skilled nursing in one instance," writes the correspondent, "soon told its tale, and I regret to say that first one child and then the mother succumbed. I have no hesitation in saying that things might have been otherwise if nurses had been available." It is obvious that the rules of a nursing association must be adhered to; and though it is easy to suggest the addition of a special fever nurse to the staff, there is the question of ways and means to be considered. Still, if there are patients suffering from scarlet fever in Shrewsbury, there ought to be some one to nurse them.

### THE REORGANISATION OF THE MALVERN NURSING ASSOCIATION.

PEACE reigns again at Malvern between doctors and nurses. The Nursing Association has been reorganised, and the very reasonable and proper demand that two medical men should be placed upon the committee has been acceded to. As Dr. Grindrod told the meeting at which the new rules were adopted and the new committee appointed, "in the opposition to the admission of medical men Malvern stood almost, if not entirely, alone. In most cases not only were medical men put upon committees, but their services were actually sought after." With Lady Emily Foley and Mrs. Heywood as patronesses, and a committee including six ladies, two doctors, and one clergyman, the association enters upon a new era of its career. We hope that it will soon be able to afford the second nurse, whose services seem to be urgently required.

### ROYAL BRITISH NURSES' ASSOCIATION.

THE annual conversazione of the Royal British Nurses' Association will be held on Monday, December 4th, at the Royal Institute of the Painters in Water-colours, Piccadilly. A very attractive programme of amusements will be provided; Mr. George Grossmith has promised to assist, and Mr. James Dunn, of the Palace Theatre, to contribute a banjo sketch. The advance model of the musical mutoscope has been lent for the occasion. The railway companies have arranged to issue return tickets at single fare from all parts of the country from December 1st to 5th inclusive. The price of a member's ticket for the conversazione is 1s. 6d., of a friend's 2s. 6d., and of a guest's 5s. Members who take advantage of the cheap travelling facilities will be able to attend the sessional lecture on Saturday, December 2nd, at six p.m. The address will be delivered at 11, Chandos Street, Cavendish Square, by Sir Richard Thorne Thorne upon "The Prevention of Consumption." Members are admitted to the sessional lectures free, and may obtain tickets for their friends on application to the Secretary of the Royal British Nurses' Association, 17, Old Cavendish Street, W.

### THE PERILS OF IGNORANCE IN NURSING.

IN the course of a lecture delivered to the graduating class of nurses at Freedman's Hospital, Washington, U.S., the Rev. Walter H. Brooks protested against "the precious life of the sick" being entrusted to ignorant nurses. Mr. Brooks mentioned that "one of the saddest deaths that ever occurred in the State of Virginia was due to the pitiable blundering of an ignorant nurse." Her patient was her husband, the pastor of a large church, and she caused his death by giving him the wrong medicine. Mr. Brooks added, "God alone knows how many lives have been sacrificed on the altars of ignorance and stupidity by devoted attendants, who, with all sincerity of purpose, put their patients to death through deeds of mistaken kindness. I dare say that thousands of people now dead would be inhabitants of earth, and in the enjoyment of health, had they or their friends called in a trained nurse in the time of their illness."

### PLAGUE NURSING AT SATARA.

A NURSE on plague duty at Satara writes: "This is a lovely little place. I was sent up here all alone. The journey from Bombay to Satara Road Station was delightful; the scenery just like parts of Switzerland. From Satara Road I had to drive in a sort of four-wheeled dog-cart, called a 'tonga,' for ten miles. We started at three a.m., and arrived here at the Lâk Bungalow, or Travellers' Rest House, about five a.m. A Chota Hazru was waiting for me; I was glad of the tea, for I had been travelling from two o'clock p.m. the previous day. Then I lay down, hoping to sleep; but at eight a.m. I heard someone say in broken English: 'Madame Sahib, wake up, getting late; Collector Sahib say he come at nine o'clock.' I stumbled out of bed, and had hardly scrambled into my dressing-gown when a *man* came in to get my bath ready. Well, it did not take me half an hour to have my bath and dress, and I was ready in full uniform to receive the Collector Sahib, who arrived at nine punctually. We had a chat, and he said that 'I had better see some bungalows which were to let, and furnish one for myself and the nurse who was comirg to join me, and that he would send some servants for me to choose from.' Very soon I was inundated with cooks, mailers, tonga-wallahs, landlords (who had bungalows to let), cloth sellers, and grocers, who overwhelmed me with offers of assistance. I went over no less than ten bungalows one morning, and finally selected this, which is partially furnished. Alas! I left all my pretty things at home, and now what would I not give to have them! The people here are most hospitable, and I get asked out to tea, dinner, drives, tennis, and golf. Yesterday the first case of plague appeared; it is a bad one, and I will tell you about it later on."

### SHORT ITEMS.

ON Saturday the Princess Louise opened a sale of work at the Queen Victoria Jubilee Nurses' Institute at Edinburgh, in aid of funds for the extension of the institute.—The committee of the De Moir Memorial Fund have decided to expend a portion of the £416 collected to the purpose of providing a cot in the Memorial Cottage Hospital at St. Andrew's.—At the annual meeting of the Glasgow and West of Scotland Co-operation of Trained Nurses it transpired that there are now 97 nurses on the roll. The gross sum earned by nurses was £5,936, an increase of £1,833 on the previous year; and the financial statement showed a balance of £296 profit.—The Jubilee Convalescent Home, which was opened at Bristol by the Queen last week, has since been thrown open to the general public, of whom thousands have visited it. It is hoped that the Home will be ready for the reception of patients at the end of the month, and the Bishop of Bristol has kindly promised to take the service in the little chapel on December 3rd.—A Shilling Nurses' War Fund has been opened at Manchester, and the contributions up to the present time amount to £8 4s. 6d. Mrs. Chapman, of Old Hall, Whitefield, is the hon. treasurer.—It is reported from Capetown that the Ambulance Corps of the Rouxville Commando, on its march to Burghersdorp, was delayed while crossing the river owing to a nurse having been accidentally shot dead.—On Tuesday evening a number of the inmates at the Brompton Consumption Hospital were entertained by Miss Italia Conti and her friends in the theatre to a performance of five scenes from Mr. Daly's comedy, "The Last Word," and the comedietta, "A Cure for Eviction." The entertainment was so much enjoyed that the patients often forgot to cough.—Mrs. Weekes—better known as Nurse David—has been presented with a handsome brass inkstand, accompanied by an illuminated address, from the people of Barnwell, Cambridge, among whom she laboured as district nurse for some time. The presentation was really a wedding gift, made subsequent to the marriage of Miss David to the Rev. C. M. Weekes.

### Lectures to Surgical Nurses.

By H. A. LATIMER, M.D. (Dunelm), M.R.C.S. of Eng., L.S.A. of London, Consulting Surgeon, Swansea Hospital; Past President of the Swansea Medical Society; Lecturer and Examiner of the St. John Ambulance Association, &c.

### DISEASE OF SPINAL BONES (*continued*)—SPINAL ABSCESSES—NURSING SPINAL CASES—CARE OF CATHETERS IN URINARY CASES.

SPINAL disease may exist from without or within. In surgery we are concerned with those cases arising from external interference—whether from injuries or from diseases of the bones encircling it. In the case of injuries to the spine, more or less complete paralysis of all voluntary movements of parts below the injured spinal cord will result—complete if the displaced bones or effused blood resulting from injury press so severely upon the cord that nerve communication between the brain and the part it desires to communicate with is shut off altogether; incomplete, if it be only pressed upon in a slighter degree. If the interference should be at a high point in the cord, where the centres of respiration and circulation of blood are situated, immediate death will follow a severe injury. This is the mechanism of judicial hanging. Here the neck vertebræ enclosing those vital centres are suddenly dislocated; they suddenly crush the underlying cord, and the actions of the lungs and heart are immediately arrested. Apply this illustration to successive sections of the cord and you will realise that complete dislocation of vertebræ anywhere will abolish function below the seat of injury.

The most common disease affecting the bones here is that caused by tubercle. This is very apt to be deposited in the bodies of the vertebræ, and, as a result of it, they fall in and cause curvatures of the spine, which are generally called after the name of the distinguished surgeon Potts, who drew attention to them. You will often hear of Potts's disease of the spine or of Potts' curvature.

In the earlier stages of this affection the child—for it is generally a child who is the patient—suffers from pains connected with the back, and is noticed to hold himself stiffly. Later on fever declares itself, and when the disease has progressed sufficiently to allow of yielding of bones or ligaments at the affected part, displacement of these generally takes place, and the back becomes curved in such a manner that it is said to have "grown out." The rate of progress in the disease may be so slow that the spinal cord has time to accommodate itself to the new positions it has been compelled to assume. In such a case the results of disease are more likely to show themselves in general exhaustion of the system by continued fever or by abscesses, which result directly from the bone disease, than by any direct effect upon the spinal cord itself. Abscesses are particularly liable to form and to show themselves at a distance from the seat of mischief, doing so here, as elsewhere, where bone is inflamed. You may have been puzzled at seeing the surgeon in attendance on such a case as this seek for the evidence of matter in front of the upper part of the thigh, and by hearing him declare, should he find it there, that the patient is suffering from a psoas abscess. By this term is meant an abscess which is contained in the sheath of a muscle called the psoas. Although matter which has formed in connection with disease of the spinal bones may show itself in the back —as a so-called "lumbar abscess"—at the outer side of the front of the thigh, at the abdomen, in the buttock, or elsewhere in these regions, psoas abscess is the characteristic result of such disease when it affects any of the bones at or below the waist. There is an anatomical reason for this fact. For this muscle has its fixed points above, at the bodies and sides of some of these bones, and then, passing downwards along the back of the abdomen, it comes forward and emerges from that cavity at the front of the thigh before it inserts itself in the upper part of the thigh-bone. So, if matter forms at the bones above this, pus is discharged into the sheath of the muscle and then finds its way by gravitation to its most dependent point where it shows itself by a fluctuating swelling. The efforts of the surgeon are directed towards the prevention of any such an untoward result as this. If he can get the case in an early stage he prevents all movements of the spinal column by putting up the patient in a plaster of Paris or a poro-plastic jacket, and he keeps the little patient as quiet as possible, while he causes his general health to be improved by good feeding, good air, and by the administration of suitable medicines. Should the illness have progressed to the stage of abscess this is opened under the greatest precautions of antisepticism, for such collections of pus as these are peculiarly liable to lead to bad results by continued suppuration and fever. Under the bolder surgery of later days the spine is sometimes cut down upon and the operation of laminectomy is performed, that is, portions of the diseased bones are removed, but in many cases it is useless to do this. If we could only get patients under treatment at the early stages of spinal disease these untoward results would often be obviated, but we often do not see them at this stage of the malady.

But it is especially in the paralytic affections following disease of the spinal cord that you will have so much to do in the way of nursing. Here all your skill will be called for in relieving the patient of a number of distressing results of his disease, and in preventing the supervention of fresh troubles which are directly the result of loss of the function of reflex action. For, owing to the centres of defecation and urination becoming blunted or paralysed, the bowels and bladder soon become a source of the greatest distress and danger. In the first case the power to expel the fæces is lost, and these accumulate in such a way that they have to be removed by enemata or the scoop; in the second, after a preliminary stage of retention of urine in the bladder, this viscus becomes overfilled, and the contained fluid then dribbles away, just as water would from an over-filled cistern. The fæcal retention is bad enough, but the incontinence of urine is far worse to deal with. It is always a matter of extreme difficulty to prevent the skin of the back from breaking down into sores—bed-sores as they are called—in cases of paralysis, for the skin there is badly nourished, and has but a poor vitality under such circumstances; but it is peculiarly difficult to ward this off if incontinence of urine prevails, for then an already devitalised part is irritated and inflamed by contact with a decomposing and peculiarly destructive fluid. The readiness with which the skin and subcutaneous tissues slough in spinal disease is due, not merely to the presence of this decomposing urine, but is the result of an interference with the vitalising principle carried to the parts below the seat of mischief by nerves. The spinal nerves possess the functions of transmitting impulses to move muscles and of receiving impressions of pain or other sensitive feeling, and, in addition to these properties, they have the faculty of providing for the life or vitality of the arcs over which they preside. This last work of theirs is known as their "trophic function," the word trophic being derived from a Greek source, and meaning "nourishing." It is by injury to this part of the nerves that the resistance of the skin to the irritating influence of the urine is sapped, and its vitality is destroyed. In such cases the bladder will be emptied by systematic use of the catheter, and the bed-sores will be prevented from forming, or will be treated, should they have formed, on the principles I have laid down in lecture 24 of these series.

### The Colonial Nursing Association.

### IV.—WHAT IT MIGHT ACCOMPLISH FOR THE EMPIRE.

Although the progress of the Colonial Nursing Association in three years has been remarkable, and it has been able, to some extent, to solve the problem of how to provide for the nursing of English people in the Crown colonies, there can be no question that the movement is yet in its infancy. Those who have eyes to see must clearly recognise the fact that it is capable of large and lasting developments. With the true prophetic instinct of a practical enthusiast who knows how to assist in the fulfilment of a prediction, the founder in her report of 1898 declared that "the association has undoubtedly a great future before it," and the more its object and the scope of its operations is realised, the more speedily will the forecast of Mrs. Francis Piggott be verified.

In dealing with its immediate needs, one of the great objects which the association might accomplish for the Empire was adumbrated. Its usefulness cannot, in fact, be extended in the manner desired so long as the state of affairs existing in many of the Crown colonies remains unaltered. Inquiry must precede reform, and until the reorganisation which the colonial hospital system requires in many places has been effected, the work of the association must be on a comparatively limited scale. In December, 1895, papers relative to the provision of nurses in the Crown colonies were printed. They embodied the opinions of a number of colonial medical officers, who were invited by the Colonial Secretary to attend a meeting at the Colonial Office to consider the matter of providing trained nurses for the benefit of European residents. Naturally, in each colony opinions varied as to the methods to be adopted. It is amusing to observe that the medical officer in Ceylon and the colonial surgeon and inspector of hospitals in Hong Kong expressed absolutely opposing views on the vexed question of nursing by French sisters. The former declared that he was "quite satisfied with the system," and alluded to it as "the best and cheapest that can be supplied." One of his arguments in favour of it was that marriage, which he looks upon as "the most disorganising element in all secular nursing systems," is out of the question. The Hong Kong colonial surgeon gave a striking illustration of the practical result of employing French sisters in the Government Civil Hospital. "On the score of economy," a staff of nine nurses under a lady superior was obtained from the French Sisters of Mercy in connection with the French convent. "The sisters were found to be only supervisors. They objected to taking notes of any directions given by the medical staff, and these directions were passed verbally from one nurse to another on being relieved; this led to misunderstandings, directions got mutilated and some forgotten, to the great detriment of patients. They also declined to take notes of temperature; they would not administer enemas or vaginal injections. They would not be present when there was any exposure of the person, even accidentally when a patient was delirious and threw off the clothes, or when it was absolutely necessary either in male or female cases. I therefore had to represent to Government that we were put to much extra expense with very little gain, as we had to retain most of the Chinese staff, and that the nursing was a failure."

This was in 1889. In 1890, five nurses, trained at the London hospitals, were obtained under a matron, and since then Hong Kong has had the honour of possessing the best organised hospital in the whole of the Crown colonies. Moreover, the Hong Kong Hospital was the first to follow the suggestion of the Colonial Nursing Association that the staff should always contain two or more nurses available for private cases outside. Two nurses were sent out in May, 1898, on that condition, and the experiment has proved the greatest boon to the colony. When they are not engaged outside, these nurses work with the ordinary staff. The idea of Mrs. Piggott has always been that the colony should invariably provide for its own Government hospital matron, a fully trained woman from England, and that where the need exists for nurses for private work the hospital authorities should supply board and lodging, and look to the fees of the nurses and to local subscriptions to cover expenses of their passage and salary, supplemented by such grants as the Colonial Nursing Association may think fit to make when the want of assistance is clearly demonstrated. The need of private nursing really exists everywhere, and it is obvious that if the suggested system were adopted it would do away with the necessity for local committees. They are very well in their way, but in the Crown colonies the hospital is the proper head-quarters of a nursing staff, and the nurses should be under the control of the Government matron. The association would still select and send out nurses, and render any financial assistance essential.

In order, however, to make the system a success, an inquiry into the nursing arrangements of many of the colonies is indispensable. In June, 1898, the Colonial Nursing Associational asked the Colonial Office for a return to the following questions: (1) What hospitals exist in the colonies? How many beds do they contain respectively? (2) Is there a nursing staff in each, or which, hospital? Of whom is the staff composed? Is there an English matron? What is the number of nurses employed, and are they all, or how many, fully trained? From what source are they obtained? (3) What provision is made in the colonies for private nursing? (*a*) by means of nurses attached to the hospitals; (*b*) by nurses not attached to the hospitals?

The Colonial Office are now considering a circular dispatch based on these interrogations, which, it may be hoped, will have the effect of eliciting the important information required. Pending this return, the Colonial Office forwarded Mrs. Piggott some figures extracted from Blue Books, which throw a certain amount of light upon the position. Thus, for instance, there are none but "attendants" employed at a considerable number of hospitals; in some of the colonies there is no hospital at all; and in several cases the only statistics given are comprised in the laconic words, "No returns." But as no answer is given in most cases to the vital question, "From what source are the nurses drawn?" the document which really supplies all the information available is not of much use. Indeed, it materially strengthens the plea for an exhaustive investigation, and tends to deepen the conviction that a vigilant commission of inquiry might discover many anomalies and defects.

It is most desirable that there should be expedition, for the issues of life and death are at stake. This has been illustrated in a striking manner by a memorandum just received upon nursing in Dominica, where, it is stated, many lives are yearly sacrificed for want of nurses. Doctors' instructions as to the regular administration of medicine, food, and diet are repeatedly not carried out because the relatives have not leisure to attend to the patient, or fail to see the need of following his directions. Even a properly trained midwife is unobtainable, and consequently there is often injury to the mother and child, as well as frequent deaths. The uneducated women, who are the only persons willing to attend confinement cases, are incompetent, superstitious, and in many other ways objectionable. The question of cottage hospitals in the up-country district is particularly pressing. It often happens that on account of the Government hospital being below fever level, or because of its insufficient nursing staff, or its absence of accommodation, patients who would be glad to pay cannot be brought down to it. While Mrs. Piggott was organising the Colonial Nursing Association in August, 1896, two middies were attacked by typhoid fever while they were on board the flagship lying in the harbour. They, of course, had to be removed, but the Government Hospital at Port Louis was out of the question because there was no accommodation. By the kindness of the officer commanding, they were carried up into camp, and a bungalow was placed at their disposal, but there being no woman available they were "nursed" by orderlies until the death of one and the partial recovery of the other. Here, by the way, is an excellent reason why the Admiralty should subscribe to the Association. The Navy is often in need of a nurse at out of the way stations. This, too, opens up in one's mind the vision of another duty which the Association might easily discharge. It has been well said that the good work done by it in the past only shows how much still remains to do. An enormous impetus would be given to its progress if continuity could be secured. Lack of continuity has been the cause of most of our failures in the colonies. One governor undoes what his predecessor has done; one medical officer changes the procedure of the man before him; and so it goes on. A settled policy is as much wanted in respect to hospitals and nursing as it is in our international relations. The policy of the Colonial Nursing Association is clear and sensible; it has been successful wherever it has been applied; and when it has been enforced in of all the Crown colonies we shall wonder how we endured the previous situation in any of them. We look forward to the time when under its ægis the sick and suffering sons and daughters of England in the most remote corners of the Empire will be as readily able to command the services of thoroughly trained nurses as the victim of an accident in a London street, or the prey to an epidemic of typhoid in a provincial watering place.

### The Eviction of British Nurses from Johannesburg Hospital.

It turns out to be a fact that the whole of the British nurses have been evicted from the Johannesburg Hospital. When the report was first circulated we hesitated to credit it, and hoped that it had reference to a minor institution. But we were mistaken. Nearly fifty nurses were turned out of the Johannesburg Hospital at three hours' notice. By the courtesy of a correspondent we are able to give the following account from the *Natal Mercury* of October 23rd :—

### The Medical Officers Tell the Story.

"Drs. Taar and Hunter, who were, until recently, senior and second house surgeons respectively in the Johannesburg Hospital, called at the *Mercury* office on Saturday afternoon and gave the following information in regard to the commandeering of the hospital by the Transvaal Government. They stated that Dr. Mangold (a Frenchman), who took over the hospital on behalf of the Government, called for that purpose at about half-past seven last Monday morning. He immediately interviewed the medical staff, shook hands cordially with them, and hoped that they would be good colleagues, as the Government were quite agreeable to allow them to continue their services. The new chief then interviewed the matron (Miss Young), and by undertaking certain things for which he had no authority, extracted a promise from her that she would remain, with all the staff."

### Dr. Mangold Orders the Nursing Staff to Clear Out.

"At half-past eleven, however, when Miss Young again saw Dr. Mangold, she told him that after consideration, and owing to several matters about which she had heard, she had decided to leave the country. Miss Young also stated that she would not influence the nurses in any way as to their going or staying, but that she would look after any members of the staff who decided to leave. On hearing this, Dr. Mangold said that he would obtain protection for nurses if they would guarantee to stay, but when asked to produce his authority for this statement he could not do so. He stated that he would obtain it before four o'clock, however, but when that hour arrived the position of affairs was unchanged, and he practically ordered the nursing staff to clear out. At the same time he informed the matron that the d——d young English doctors would have to do as they were told, or be hanged."

### Two of the Doctors Go Also.

"Meanwhile the names of the whole hospital staff had been put on the provisional permit list, and they received their permits in the course of the day. That same afternoon the resident medical officer, Dr. Peirce (a Free State burgher, but a Natalian by birth) warned Dr. Taar that he had better be very careful as to what he said, as Dr. Mangold had stated that he (Dr. Taar) had been using treasonable language, and that Dr. Hunter had also been showing his English sympathies too freely, and had told the nurses that they were quite right to clear out. By this time both doctors had got permits, and had taken the oath of neutrality, but in spite of this fact they saw trouble not far ahead, and accordingly decided to leave for Durban, viâ Delagoa Bay. In their own words, 'We were foolish enough to buy railway tickets,' and getting on the train travelled to Delagoa Bay in coal trucks. Arriving there they succeeded in booking a passage in the 'Raglan Castle,' and came to Durban on Saturday along with the nurses. The latter left Johannesburg on Tuesday night, and on reaching Komatie Poort the carriages in which they were travelling would have been shifted into a siding, and probably left there, had it not been that the guard had promised Commandant Schutte to see the ladies safely through, and owing to the state of matters it was fortunate that the promise had been made."

### Indignation of the Officials and Public.

"The Customs officials at Komatie Poort were very indignant when they learned that the nurses had been ordered to leave Johannesburg, and stopped the usual searching on the facts becoming known, as being the only way available to express their sympathy. The people of Johannesburg were also highly indignant at the treatment meted out to the nurses, and showed practical sympathy by subscribing £800 to pay their travelling expenses to Durban, &c. Before Miss Young and her staff left the hospital, Dr. Mangold had received a telegram from President Kruger ordering that all nurses should leave, except Colonials. This was, of course, the last straw, and out of a staff of forty-six, forty entrained for Delagoa Bay—those left being either of German or Dutch extraction."

### The New Nurses.

"On Monday afternoon Dr. Mangold was good enough to tell Miss Young that she and her staff could go, as they would be replaced by ladies from the most aristocratic families in the Transvaal, including his own wife. In connection with the latter statement, it may be noted that a few doctors in Johannesburg had lately been giving a course of ambulance lectures to Boer ladies, and it is these ladies—with the knowledge imparted in twelve lectures—that have taken the place of trained and experienced nurses. The thought makes one feel sorry for the wounded and sick inmates of Johannesburg Hospital."

### Louise Darche: A Modern Nurse and Her Work.

The following interesting appreciation of the late Miss Darche is from the pen of Miss L. L. Dock:—Shall we always look into the past for examples of our great ones in nursing reform work? We read the lives of Florence Nightingale, of Sister Dora, of Agnes Jones, and thrill over their struggles and achievements. Shall we have read these lives aright if in idealising them we overlook those who are working here—to-day—by our side—in our midst? Are we any better for studying and for revering them if in dreaming of these heroines of the past we neglect to see that here, toiling before our eyes, is Florence Nightingale; there is Sister Dora; there is Agnes Jones, differing only in outward appearance and in the setting and surroundings of their tasks? To my mind it is so refreshing to see what people are doing to-day! So beautiful to know the ideal under the prosaic exterior of our associates in daily life! To this end I would try to bring out something of the story of one, lately amongst us nurses, whose work in its isolation, its heaviness, its peculiar difficulties took second place to none, and whose entire devotion to her task made her truly a living sacrifice for its sake. I mean Louise Darche, for ten years associated with the work of nursing reform, hospital management, and civil service reform on Blackwell's Island, New York City.

### What She Had to Struggle Against.

The condition of our municipal politics is so well known by all intelligent people that I need not attempt to describe the setting of the scene of her labours: the great city hospitals infected with every form of moral rottenness, usurped and controlled by men who had no standards but self-interest and greed while doing public work which was a public trust, and demanded the deepest sense of human obligation. The work she was called to do was not only nursing; it was a continuous struggle against evil in the concrete form of selfish and unprincipled men, who turned to their own bad ends the provision made by the city for its suffering poor. But no less formidable than the enemy she undertook to face were the qualities of intellectual and moral strength with which this nature of rare sincerity and courage was endowed. With these weapons alone did she many and many a time confront, daunt, and defeat those whose constant aim was to pull down and crush the principles of truth and justice which she upheld. Truly, she did noble work in the reform of nursing and hospital management; but besides this, as a servant of the great city she was a true civil service reformer, worthy to stand in company with the most distinguished of those men who have fought, and are yet fighting, the same battle which she fought for the triumph of the principle of "merit" over that of "influence" or "pull."

### How She Came to be Appointed to Blackwell's Island.

It has been sometimes supposed by those not acquainted with the inner history of the island that Miss Darche's appointment, having been made by and under the Commissioners of Charities and Correction, she was, therefore, but a political or, at least, semi-political appointee. But nothing could have been more erroneous. Her appointment was the result of one of those long, arduous, and most patient struggles for better conditions made by good women and men—the State Charities Aid Association of New York City—in the interests of the sick and afflicted poor. As the result of their reports made year by year, the time came when their recommendations were heard and the Commissioners stood ready to appoint the right woman to the head of the nursing on Blackwell's Island, should the women of the State Charities Aid succeed in finding her. They went to the superintendent of the training school for nurses attached to Bellevue Hospital, Miss Perkins, who chose Miss Darche for the work and persuaded her to take it. The situation was a most delicate one. Nominally appointed by the Commissioners, she was really placed in their midst by the moral courage of a few women who voiced a public conscience absolutely opposed to the aims and standards of political parties; and for the accomplishment of the practical reforms she was meant to make she had to win daily from these men, by tact and skill, their consent to arrangements which were a tacit condemnation of their own methods, their signatures to measures which were an attack on and an undermining of their own political jobbery, and often their protection and upholding of herself in her province of authority in nursing matters and ward management against their very own underlings, to whom they gave adherence and from whom they exacted strict allegiance.

I will not now describe the conditions that prevailed in the Island hospitals when Miss Darche began her work there. They were the same that Agnes Jones found in the great infirmary in Liverpool; that Miss Fisher found in Blockley; that Sister Helen found in Bellevue. The women of our profession who have worked in similar places do not need to be told, and others would not understand—perhaps would hardly believe. Then, too, if they wish, they may refer to the reports of the State Charities Aid.

### The Friends Who Helped Her.

Amid the most repulsive surroundings, but cheered and strengthened by the companionship of a friend, Miss Darche took up her load. During the ten years in which she gave herself unstintedly, mind, heart, and soul, to her work, she had always the warmest support and encouragement of the members of the State Charities Aid Association. Especially did she rely on the unfailing sympathy and ever resourceful help of Miss Rosalie Butler (while she lived) and Mrs. Cadwalader Jones. They were not only personally kind; they were also powerful allies, without whose support no woman could have kept such a position and yet have preserved, as Miss Darche did, perfect freedom from political truckling. But, though they were strong and skilful, they could not do her work, nor could they have maintained her there had not her own keen mind and gift of generalship, such as many men might be proud to possess, enabled her to hold her own with the commissioners and the long list of minor officials attached to a large city hospital.

She used no flattery or feminine wiles such as women are too often driven to resort to. Hers was a master mind, and she had an unerring capacity for gauging human nature. From the better ones of the natures about her she won her points, slowly, one by one, by confidence in their better impulses, by a rational and unanswerably logical presentation of her subject, or by the sheer force of her own moral strength. For the meaner opponents, who met her with lying and intrigue, she had a marvellous skill in turning their weapons against themselves to their utter confusion. While many such hated her, not one but was compelled to respect her.

### The Merit System.

Miss Darche's foremost and constant purpose was to create and maintain in her school that merit system, the bed-rock of our training schools for nurses, without which no good work can be done. Her determination was inflexible to permit no slightest infraction of this principle, knowing that its enforcement was the only security for good nursing, for the care of ward and hospital property, for that moral refinement which is the greatest change good women have brought into hospital work, and that it was the first essential toward inducing women of a high type to enter the school. This rule was to her the test by which all else must be tried. To break it was to commit the unpardonable sin, and during all her ten years of life on the Island she held herself ready at any moment to resign her position rather than yield an iota in this respect. Failure in this point would, she felt, have meant that her prestige was gone. All else she bore and patiently strove under, believing that time and effort would set all things right. The establishment of the merit system had the effect of a revolution in this school, heretofore degraded to the level of providing places for the friends of small politicians; but it is one of her most brilliant achievements that she never did make the smallest sacrifice of her integrity in this regard. On one occasion notably she was victorious, although the invader was no less a person than the Mayor of the city himself, who desired to have an unfit appointment made among the nurses for political reasons. She refused, and the Mayor in his wrath was indiscreet enough to complain aloud that he could not get his friends into the Island hospital. His complaint reached the papers of the opposition, and thence the public, to his discomfiture and not to Miss Darche's injury.

A Nurse's Home Without Home Comforts.

The changes and improvements made in the nursing service during those ten years can hardly be told in a short article, and they are outlined in the school reports. When Miss Darche and her friend went to the Island they found a Nurses' Home dreary as barracks, with insufficient accommodations, wretched dining-room service, and no home comforts. Prisoner women of the most degraded type did the housework and filled the halls with ribaldry. There was no systematic course of study for the nurses and no lectures. The pupils worked in the Maternity Hospital and in the wards of Charity Hospital, which received chronic cases alone and had no "all round" training. Yet there were among them some excellent women, whose good qualities Miss Darche was the first to appreciate and utilise to the utmost. Here might some equally zealous but less sincere reformers take useful points from her, that even under a bad system she did not prejudge every individual as bad, but searched for the good element that it might be given its just dues and be raised to a higher efficiency. The first work in the home was to get rid of the prisoner women and establish a decent service. The building itself was fine and beautifully situated on the point of the Island. Now for many years the nurses have enjoyed as comfortable and refined a home as may be found in any hospital. The large parlours are filled with books, many of which were given by Mrs. Cadwalader Jones, ever generous and sympathetic, and other friends of the school and of Miss Darche, among them Marion Crawford, who gave Miss Darche's surname to one of his heroines, and who presented the school with a complete set of his own novels.

A study room was fitted up with technical books and magazines, where teaching by demonstration was carried on, and a course of lectures each winter was provided by the ladies of the State Charities Aid Association.

Florence Nightingale's Letter.

In Miss Darche's office hung framed an autograph letter written to her by Florence Nightingale. They were personally acquainted, and in two visits made to the venerable mother of nursing Miss Darche had been assured of Miss Nightingale's kindliest interest and well-wishing in her work.

Improvements in the Home and School.

Important extensions were made to the home, planned and superintended by Miss Darche, and costing the city almost nothing, by which the main building was connected with some old and disused wards. These, repaired and renovated, made a delightful wing and allowed for that enlargement of the school made necessary through increasing work. One by one three other hospitals belonging to the city—the three emergency hospitals, Gouverneur, Harlem, and Fordham—were taken under Miss Darche's charge, making five separate institutions to which the nurses had daily to be sent, and requiring five times the amount of planning, thought, and care which is usually demanded of the head of a hospital nursing corps. The number of patients daily cared for averaged 850.

What Miss Darche Aimed at.

From an early period her clear, logical intellect had grasped all the conditions of the city institutions, and she ardently desired to see them brought into an orderly and unified organisation. Her whole conception, simply presented in its main outlines, was as follows: That a nursing department might be created under the city charter, under one head who should be a trained nurse, and that this department should take charge of all the nursing and housekeeping or domestic service throughout the city institutions; that the training school on the Island might, as heretofore, train nurses, sending them in rotation to those institutions which gave a complete service; and that such institutions as the almshouse, the asylums for youthful and for adult imbeciles and feeble-minded, the babies' hospital on Randall's Island, the refuges for older children, and the contagious hospitals should be nursed by permanent staffs selected from the graduates of the school, who might be attracted to these lines of work by comfortable surroundings, graded salaries, opportunities for promotion, and the right system of organisation and responsibility. This plan included, of course, responsible matrons and head nurses at the head of each institution, on good salary and responsible to the head of the nursing department.

Outward Protection.

The main purpose toward which she unceasingly bent her mind and energy was that of securing for the school some outward protection—perhaps a commission, formed of members of the State Charities Aid Association or other public-spirited citizens, whose powers should be more than simply advisory, and who would be a bulwark against political aggression. As reform was promised and the election of Mr. Low seemed likely, she believed that the attainment and fruition of her long and exhaustive endeavours was at hand. Mayor Strong had been her staunch friend and protector, but while reform was in the experimental stage not all could be immediately accomplished. Eagerly she looked forward to the possibility of establishing on a firm basis the work which she longed to lay down, for it was fast becoming too heavy to be borne, but which, while it was yet unfinished and hope remained, she would not desert.

The End.

When finally Mr. Low was defeated, and all further effort was seen to be useless, the reaction was such as strong men have gone down under, when in one moment they see the ruin of all their lives have been spent for.

Her friends feel that here, indeed, was an untimely sacrifice of a noble nature; that here was lost a true soldier, who fought in the very thickest of the fray the ever recurring battle of high ideals against baseness. It would be hard, indeed, to feel that such sacrifice had been made in vain!

I have not attempted a character sketch in this outline of the work of a modern nurse, save in so far as what she was is expressed in what she did and aimed at doing. I would not have her appear faultless, for none are so; yet this one word for friendship's sake. All who met Miss Darche must have felt her candour. All who worked with her knew her justice and uprightness. But only those admitted to her intimacy knew all the sweetness, the fidelity, the clear simplicity that lay beneath.

Shall we, then, look only into the past for our ideals? Rather let us give to those about us, while they yet live to need it, the comfort and strength of our approbation and sympathy.

### The Opening of the Bristol Jubilee Convalescent Home.

By One of the Nurses.

Though all Bristol was busy preparing for the Queen, inside the Convalescent Home the scene was busiest of all. Her Majesty would not see it, but still everything must be in apple-pie order, to befit a place which she honoured by opening. So floors were swept and scrubbed and polished, some ninety beds were made, flowers were arranged, and ornamental plants disposed in advantageous positions. Half the entrance hall became a bank of beautiful orchids, palms, chrysanthemums, &c., piled up almost to the ceiling. On the Monday and Tuesday preceding the Royal visit the workers had a pleasant pause in their labours while the Royal carriages were paraded in the grounds, and the proceedings of Wednesday—the day—were rehearsed. We all got up early on Wednesday to put the finishing touches. It was a great relief to see the morning was fine, for the stormy weather of the previous week had made us fear we might at the last be disappointed of seeing the Queen; but as usual her Majesty was favoured. Then, new uniforms were donned, and long before the appointed hour, the large stand at the back of the house began to fill. All sorts and conditions were there—Church dignitaries, city magnates, Town Councillors, great people and small—all eager for the best place to see the Sovereign. The band of the Life Guards enlivened us with music, and helped to make the time pass pleasantly. It also prevented us from hearing the Royal salute fired near by on the Downs when the Queen reached Bristol station. Nor could we hear the voices of the 27,000 children singing to the Queen, though they were not many hundred yards away.

But at last, by the arrival of the Lord and Lady Mayoress, we knew that the supreme moment was at hand. Then the Royal liveries were seen, and the three Royal carriages which preceded the Queen's drove past us, and the National Anthem struck up. I believe we stood on our chairs; I believe we sang "God Save the Queen"; I believe we cheered as we had never done before; but it was all nothing beside the fact that our dear Queen was there, and that we were close to her. I know when we sang the beautiful Jubilee hymn, and especially when we came to the second verse, we felt as if we were trying to tell her of all our love and devotion, our gratitude to her for her never-failing sympathy with her people in all their troubles and griefs. Some of us, anxious now for dear ones in South Africa, knew that she too was sharing our anxiety, and the knowledge drew us closer to her, and we put all our hearts into our singing. But that, too, passed, and then there were presentations, that of our matron, the most important to us, and all too quickly the carriage began to move off, and we, the nurses of the home, had to lead the larger crowd of nurses from the Royal Infirmary and the General Hospital in a rush through the house to be behind the great front door when the Queen touched the button. As the door flew open, we trooped down the steps in time to see the blue velvet case containing the button presented to Her Majesty, and then the ceremony was over and the Queen's carriage moved off. Some of us ran to the entrance gates to have a last view of her as she drove off surrounded by her escort of Life Guards. I think we should have felt terribly flat afterwards but for the crowds who have visited the home on that and every succeeding day. We hold money boxes to defray the many unforeseen extra expenses. We have collected over £116 in these boxes in the four days the home has been on view, and it is touching to see the eagerness of hardworking men and women to contribute their mite to the memorial of the Queen's Diamond Jubilee, to hear the many inquiries for the door which the Queen opened, the place where she made her mark, &c. Many stories are told us by mothers of the numbers of their little ones who sang to the Queen, and of how she seemed touched by the sight of the children! Some fancy they have been individually bowed to. One crippled man—perhaps one of the humblest of her Majesty's subjects—quaintly remarks that when he took off his hat the Queen did obeisance to him! We hear of a little blind boy who asked, "Did the Queen see me?" One woman says, "It is wonderful how lovingly everyone speaks of her." In fact, we were always loyal, but we are ten times more loyal now. The home seems to please everyone. One of our many thousands of visitors says, "It is just like a palace," and many ask how they can get a chance of coming here. We all feel proud that a place, which will be a benefit to many, will always be associated with the name of Queen Victoria. God bless her!

### The Dangers of Nursing Typhoid Fever.

From a Nurse's Point of View.

When a nurse gets typhoid fever in a good hospital where everything is up to date, there is generally an impression among both her comrades and the doctors that the nurse has not been quite careful enough in her care of herself. This is, alas! too frequently the truth. Were nurses to bear in mind the duty they owe to their hospitals to take precautions to guard against becoming patients, the mortality among them from typhoid would be considerably lessened. Night nurses are generally the victims, perhaps because their meals when on duty are not of so substantial a nature as those of the day nurses. As a rule, they have to prepare their own repasts, and sister is not always present to keep her watchful eye on them. If on night duty they should be doubly careful to take all the precautions they are told to. Nurses having charge of typhoid cases should regularly take outdoor exercise, if only for a half hour. The morning work of enema-giving should never be done on "a cup of tea." Coughing in the ward should be avoided. Fluffy fringe holds germs and should be discarded, at least while nursing typhoid or, in fact, any form of infectious disease. After attending to the patient's personal wants, the hands and forearms should be washed at once with plenty of soap and water and the nail brush. Nurses who bite their nails should not be allowed to take typhoid work. The use of the handkerchief is another matter of importance. I have known a nurse to use her handkerchief at her patient's bedside when her hands were thoroughly septic, the result being, of course, that the handkerchief became septic before being put into her pocket again. Nurses cannot be too particular about minor precautions. They should avoid breathing through the mouth when attending to a bed-pan, and if a nurse has a very septic case she would do well to keep her own bowels in good order. This is a thing that nurses frequently neglect. If at night a woollen shawl is worn in the ward it should be left behind, not carried into the bedroom. Chapped hands are a distinct means of conveying infection. Now the winter is here nurses should see that their hands are well oiled, or some aseptic ointment used after each washing of them. No linen which has been employed for personal wants of a patient should be put back in the locker, however slightly soiled. Fatal results might follow to the person who took it out for second use. Flowers, if allowed by the medical men in a typhoid ward, should be burnt and thrown away. If a nurse feels squeamish and wants to take her own temperature, she should not use her patient's thermometer.

### Echoes from the Outside World.

### AN OPEN LETTER TO A HOSPITAL NURSE.

It is pleasant to read of the Queen making arrangements for spending the early spring of next year in Bordighera. She never, I believe, makes any arrangements to be absent from England without consulting her Ministers, and, therefore, the mere announcement of her having taken the east wing of the Hotel Angst carries our thoughts forward once more to a blessed time of peace. The Queen would never go abroad for rest and enjoyment whilst her people were in trouble, not only because we could not spare her, but because her heart is so wrapped up in her subjects and their welfare, that absence at any important crisis would give her no pleasure. Probably the reason why she allowed the remonstrances of those who are anxious that she should run no risks to go unheeded, and in face of all opposition, visited Bristol last week, was because she knew her people would especially like to see her just now so that they might feel how fully she was in sympathy with her nation. Later comes the announcement that Lady White, who previously received a private letter from her Majesty, had the honour of being received on Sunday afternoon at Windsor, and that the Queen is herself desirous of sending a Christmas present to every one of the soldiers who are fighting for her, including the men of the Natal contingents and of the Colonial forces in South Africa. It was a little difficult to know what to send which would be acceptable alike to all the men, but after much thought and consultation she decided upon presenting tin chocolate specially packed. Only those who have had to undergo great fatigue upon small rations know how sustaining and acceptable chocolate can be either eaten raw, or made into a beverage. Each parcel will contain half a pound's weight, and the box will probably be always kept as a memento. The lid will have a red ground with a large gilt medallion of the Queen in the centre, as well as the Royal monogram in red, white, and blue, with the words "South Africa," 1900. On completion of the contract the dies will be broken.

The visit of the German Emperor has no effect upon us in London beyond the feeling that we are glad he feels sufficiently friendly in these troublous times to care to come over and stop with the Queen. I expect that there is some especial reason, political or otherwise, why the Emperor considers it is wiser to fight shy of the capital; but it is a great disappointment to many who would like to have had a little distraction from the sad thoughts which are naturally engendered by the war. The dinner party in St. George's Hall, Windsor, on Tuesday evening, with 150 guests, the ladies resplendent in splendid frocks and wearing glittering diamonds, the men all more or less decorated with ribands and Orders, and the whole place gleaming with the gold plate—of which about £2,000,000 worth was on show—must have been a most imposing ceremony, especially as the building was lighted up by electricity for the first time. The officials at the Castle must just now be "late to bed" and "early to rise," for the German Emperor generally transacts his official correspondence, goes for a brisk canter, and is back again for breakfast by nine o'clock. Truly, the Royal Folks work hard.

There was a vacant place at the Queen's banquet to the German Emperor at Windsor on Monday. The Prime Minister's absence was due to the death of his wife. The event was sudden at the last, but the Angel of Death had been hovering over stately Hatfield for some time. It was never expected that Lady Salisbury would recover from her illness, though Lord Edward Cecil, now shut up at Mafeking, hoped to see his mother alive again. The whole nation, without distinction of party, sympathises with Lord Salisbury and his family in the irreparable loss they have sustained. There is some anxiety lest the bereaved husband of forty-two years may be tempted to withdraw from the cares of State. Not so, it is the universal hope, will he construe his duty. In the hour of his desolation he will remember that England still has need of him.

There is nothing definite to tell you about the war, though it is quite possible, and even probable, that before this reaches you a decisive blow may have been struck by the large detachments of troops now marching up country. Ladysmith still holds out, and a private letter speaks of the provisions which were accumulated before the Boers surrounded the place being sufficient for four months' feeding of all the inhabitants on full rations. So that there is no reason why anxiety on account of the beleaguered British should be acute at present, for it appears that the Boers daily get more tired of sending shells into a place which they seem unable to seriously damage. I am especially interested in "Dusty little Ladysmith"—as it is called—because the two cousins, who have hitherto been my correspondents, have, according to letters by the last mail, gone up as volunteer nurses. Nothing more can be heard from them until the siege is raised, but then they will have heaps of interesting items to relate, for they have, I believe, to feed themselves—no unimportant item when one thinks of bread at three shillings a loaf! Mafeking and Kimberley seem, too, quite able to hold their own. The spy, Nathan Marks, is still our prisoner, and no news of further threats of the execution of the six British officers has reached us, but it is a matter for regret that scurvy has broken out at Pretoria amongst the British captives. The disease is attributed to the tinned food being of an inferior quality; and as probably the Boers have no other to offer it is difficult to see how things can be improved. On this point I imagine that Mr. Winston Churchill, who, thanks to his own pluck in defending the armoured train, is one of the prisoners of war, may hereafter have something to say. Mr. Evelyn Ashley, the biographer to Lord Palmerston, stated at Romsey on Tuesday that President Steyn had been bribed to persuade him to join his Orange Free State forces to the Transvaal Boers. This has been known for a long time as a fact by many residents in South Africa, but the sum now mentioned, £60,000, seems a big one. Of course, if Mr. Steyn had not put faith in the statement made by Oom Paul, that it would only be a victorious walk over to Durban, and that the "redcoats" would never stand up to fight, &c., &c., he would most likely have refrained from sacrificing his country and his men; but it is too late to draw back now, and I fear that he, like President Kruger, must be considerably "worried."

I have just been to a Book-title Tea, and as a good many of you must have heard of similar entertainments, but perhaps have no idea exactly how they are managed, a few particulars may prove interesting. As soon as you receive your invitation, set to work and think of a title which will lend itself aptly to illustration; say, a picture of a man with the word "married" written underneath, which stands for "Married Beneath Him,' by James Payn, or a crocus bulb, which would represent "Cometh up as a Flower," by Rhoda Broughton. If possible try to hit on something which has a pun or a joke in it. This makes so much more fun. Then attach the picture or emblem to a piece of paper, and after you have removed your jacket at your hostess' house, pin the paper on to your dress in front before entering the drawing-room. Next comes the guessing, but you are not allowed to speak your ideas aloud. You are provided with a pencil and a card, upon which is written the list of the names of the guests present, and a space left opposite for you to fill in the title of the book you fancy they represent. A few questions are allowed, but nothing of a very leading character. When ideas begin to flag the hostess generally suggests an adjournment for tea, which can be handed round in the drawing-room, though if another room is handy it is more comfortable to use it for the refreshments. In the event of a great many titles remaining unsolved after tea, the authors are sometimes called for, and this usually upsets a lot of calculations, proving conclusively that many ideas were wrong. It is customary to award a small prize to the guest who discovers the largest number of titles, and sometimes one is added for the best or the worst representation—the latter in either case to be decided by vote.

### Everybody's Opinion.

[Correspondence on all subjects is invited, but we cannot in any way be responsible for the opinions expressed by our correspondents. No communication can be entertained if the name and address of the correspondent is not given, as a guarantee of good faith but not necessarily for publication, or unless one side of the paper only is written on.]

### DISTRICT NURSES.

"One who Sympathises" writes: I am glad to see a letter from "A District Nurse" concerning the accommodation provided (or what should be) for district and parish nurses in general. My experience during the nine years I have been district nurse and midwife is that all, or nearly all, committees are so anxious to obtain a good and competent nurse for their poor that they quite forget, or utterly ignore the fact that a nurse after a long day's tramp, in all kinds of weather, really needs a quiet and comfortable home to go to. I am glad to say I have just been able to convince my committee of the necessity of this, and though the funds will not allow of providing rooms or cottage, still they are willing to furnish for me so that I can at least have quiet and a place to myself.

### THE NURSING OF TYPHOID FEVER.

"Sister Ellen" writes: I am glad to see the article on "Nursing of Typhoid Fever" in The Hospital of this week. I have been much surprised and grieved to read of frequent deaths in our ranks from the nursing of typhoid. I think it quite time we asked ourselves the reason why such catastrophes happen in our enlightened days of nursing the sick. My own opinion is that sufficient care is not exercised by the nurse, especially when such cases are in a ward of a general hospital rather than in one devoted to fever. Possibly she is not instructed upon the desirability of attention to small details. Also that nurses young in nursing experience and age should not have charge of such cases. I notice that those nurses who have succumbed to the disease are usually about twenty-two years of age, so that their youth is probably the cause of susceptibility to infection. If it be imperative to place a young nurse in charge of typhoid cases she ought to be in good health, and not run down by recent nursing; she should also be reminded not to grow careless in disinfecting her hands to the conclusion of the patient's illness and convalescence.

### WORKHOUSE NURSES AND THE BATHING OF WORKHOUSE PATIENTS.

The Superintendent Nurse of the Union Hospital, Sunderland, writes: I was suprised to read in your columns an account of how a nurse in a workhouse hospital had been dismissed from her post by the workhouse master for refusing to bath a male patient under her care. Surely the nurse did not refuse to blanket bath a male bed-ridden patient, because a properly-trained nurse would recognise that office as one of her personal duties. Or was it a fact that the nurse was expected to accompany the male patient into the bath-room? Then the Board of Guardians of that union workhouse, in sanctioning the dismissal of the nurse, have acted in an unprecedented manner. It is the medical officer of the workhouse who directs when his patients may go into the bath-room to be bathed, and it is the duty of the master of the workhouse to find a male officer to attend and perform the office of bathing the male patients; or, where the Board of Guardians have not seen the necessity of appointing someone to this important vigilance, the workhouse master is called upon to send an able-bodied inmate to perform the ceremony. At the same time it is usual to caution the nurse to prepare each bath herself, and to remain near the bath-room so that she may be in readiness should an accident occur or the patient be taken ill, or should she hear the patient being roughly used. It will be another great step in modern pauper nursing when the Guardians of a union workhouse will see fit to appoint anyone but a brother pauper to take the responsibility of bathing a convalescent patient or inmate in the able-bodied department. I suppose the Guardians or the workhouse master would be held directly responsible for any serious disaster which might happen to a male patient while in the bath—certainly not the female nurse. I only hope that the disagreeable occurrence complained of will come under the notice of the Local Government Board for the good of all officials concerned in the matter.

### SHOULD NURSES SMOKE?

We concluded that the interest in this question had ceased, but "C. H." sends us from the Straits Settlements the subjoined communication: I have followed this correspondence with a good deal of interest, and hope that room may be found for my opinion. Living in the East, for the most part alone, I must own to having become an habitual cigarette smoker, and I find that heat, mosquitos, and at times constant attendance on patients, all made me fly to the "fragrant weed." My motto is, "Live and let live," and surely we are all at liberty to take our recreation in the form that suits us best. In my own case, a cigarette and a book go far more towards soothing ruffled nerves than any "walk in the open air," which the heat here renders impossible. That it develops into a habit I am quite willing to admit; but I strenuously deny that when occasion demands one cannot have strength of will sufficient to lay it aside for days together until such times when duties relax, and the habit is resumed with all the more pleasure. I have never once found any of my patients objecting to my ministrations on the plea that "I reeked of stale tobacco." I indignantly disagree with the remark that smoking tends to "loose" talk. A woman, no matter what class or profession she belongs to, if she be inclined to "loose" talk as a recreation, will indulge in it whether she be a cigarette smoker or no, and I fail to see that our sense of right and wrong should be influenced, or that we should be classed as unwomanly because we find pleasure in a recreation which up to a short time ago has been exclusively appropriated by man.

### LARGE AND SMALL HOSPITALS.

"M. H." writes: I have been somewhat comforted to see amongst the correspondence a kind word for the small hospitals. I think the great ones of the nursing world have been rather inclined to sit on them indiscriminately. I do not speak without knowing, as I have had experience in two large hospitals, spending four years in the first where I was trained, and for which I have still an old regard. But this was years ago. I have long since taken up a position in one of the smaller institutions where life has been so busy and so full of interest that I have almost forgotten I may have a duty to perform towards the many nurses who have gone out from these hospitals. It is not right that they should be ignored. Why do we set such value on size? Should not quality rather than quantity be our guide? Is it right to condemn all small hospitals without knowing whether such condemnation is just? Why cannot as good work be done in a hospital with, say from forty to sixty beds, as in a larger one? The diseases and accidents to which humanity are subject are distributed with a fair amount of impartiality throughout the United Kingdom, and so afford each their allotted portion for treatment. The time is past when all serious operations were sent away to our great cities. There are doctors in the provinces who are as well able to cope with the most critical, and able also to train probationers. I have often heard it remarked, and have myself noticed that nurses trained in the smaller institutions have a better practical knowledge of their work. In the large clinical schools the probationer's duty is to fetch and carry, stand out of the way, and then clean up; whereas, in the non-clinical hospital she has to do all this, with the exception of the stand aside. Here she has an opportunity of seeing all that goes on, and of doing her share when required. The lectures which are given are for her own benefit, not over her head. We give our nurses three years' training and regular courses of lectures by the medical staff in the usual subjects. So far, those who have completed their training and obtained certificates have had no trouble in finding good appointments, but this may not always be so if we allow remarks which I have read to pass unanswered.

### For Reading to the Sick.

FOR the invisible things of Him from the creation of the world are clearly seen, being understood by the things that are made, even His eternal power and Godhead.—*Rom.* i. 20.

The works of God above, below,
  Within us and around,
Are pages in that book, to show
  How God himself is found.

The glorious sky, embracing all,
  Is like the Maker's love,
Wherewith encompassed, great and small
  In peace and order move.

The Moon above, the Church below,
  A wondrous race they run;
But all their radiance, all their glow,
  Each borrows of its Sun.

The dew of Heaven is like Thy grace,
  It steals in silence down;
But where it lights, the favour'd place
  By richest fruits is known.

One Name, above all glorious names,
  With its ten thousand tongues
The everlasting sea proclaims,
  Echoing Angelic songs.

The raging fire, the roaring wind
  Thy boundless power display;
But in the gentler breeze we find
  Thy Spirit's viewless way.

Two worlds are ours; 'tis only sin
  Forbids us to descry
The mystic Heaven and earth within,
  Plain as the sea and sky.

Thou, Who hast given me eyes to see
  And love this sight so fair,
Give me a heart to find out Thee,
  And read Thee everywhere. Amen.

—*Keble.*

**Reading.**

Man could never have framed a name for God, unless nature had taken him by her hand, and made him see something beyond what he saw, in the fire, in the wind, in the sun, and in the sky. He spoke of the fire that warmed him, of the wind that refreshed him, of the sun that gave him light, and of the sky that was above all things, and by thus simply speaking of what they all did for him, he spoke of agents behind them all, and, at last, of an Agent behind and above all the agencies of nature.—*Max Muller.*

God has created the heavens and the earth and the sea, and has most wondrously and beautifully adorned all nature, that men may see in it their mirror and their pattern. And the visible world is as it were one great holy book, full of pictures, and parables, and analogies, and other instructive things. All the things we see have their own beautiful and deep meanings.

The beautiful white clouds, the rosy sunset, the storm-winds, the soft breezes laden with the fragrance of the flowers on a spring morning, the summer sun glowing like a fiery sea, and the silent sparkling of the stars on a winter night, the mighty thunder, and the gentle chirping of the cricket on the hearth; all these things mean more than we can hear with our ears or see with our eyes.

And the dark mountain forest, and the tall oak trees, the poplars by the stream, the hawthorn hedges, the vineyards and the cornfields, the wild flowers and the clover fields, the friendly violet and the fragrant rose; all these are not there only for our use and pleasure; they are the letters of a wonderful secret writing, written by God Himself, and the thoughts of God in them.—*Stolz.*

A voice is in the wind I do not know;
A meaning on the face of the high hills
Whose utterance I cannot comprehend.
A something is behind them; that is God.

—*George McDonald.*

### Appointments.

ROYAL HALIFAX INFIRMARY.—Miss James has been appointed Matron. She was trained under the Nightingale Fund, St. Thomas's Hospital. She has since been day and night nurse in the medical and surgical wards, sister in the medical ward, sister in charge of St. Thomas's Home for Paying Patients, nurse and sister in operation theatre, sister in casualty and out-patient department, and matron's assistant and sister housekeeper—all at St. Thomas's Hospital.

CORPORATION HOSPITAL, GRIMSBY.—Miss F. Humphries has been appointed Matron. She was trained at the Royal Hospital for Diseases of the Chest, City Road, London, and the Royal Hants County Hospital, Winchester. She then joined the London Association of Nurses, 123, New Bond Street, and has been temporary matron at Grimsby Corporation Hospital for the past four months.

COTTAGE HOSPITAL, BUDLEIGH SALTERTON.—Miss Beatrice A. Sharp has been appointed Matron. She was trained at the Royal Infirmary, Hull, and has since been sister of the accident ward and theatre, Taunton and Somerset Hospital; sister of the medical wards, Jaffray Hospital, Birmingham; and for nearly six years matron of the East Cornwall Hospital, Bodmin.

BETHNAL GREEN NEW INFIRMARY, CAMBRIDGE HEATH.—Miss Joanna Elizabeth Hopper has been appointed Matron. She was trained at the London Hospital, and has since been sister at Taunton and Somerset Hospital, matron at Sidmouth Cottage Hospital, and matron at Leeds Union Infirmary.

BRENTFORD COTTAGE HOSPITAL.—Miss H. A. Lucas has been appointed Nurse-Matron. She was trained at the Seamen's Hospital, Greenwich, and holds the L.O.S. certificate. She has since been night sister at the Seamen's Hospital, and nurse-in-charge of a private hospital in British Columbia.

SHOREDITCH INFIRMARY.—Miss Annie Oliver Appleyard has been appointed Assistant Matron. She was trained at St. Bartholomew's Hospital, and has since been night sister and assistant matron at Chelsea Hospital for Women.

### Presentations.

EAST POORHOUSE, ABERDEEN.—Last week the nurses and officials of this institution met in the board room to present Nurse Agnes McGregor with a very handsome gold-mounted umbrella and a hand-bag on the occasion of her leaving to fill the post of first assistant nurse at the North-Western Hospital, Hampstead. Nurse McGregor was trained three years in the hospital of East Poorhouse, Aberdeen, and she carries with her to London the good wishes of all her fellow workers.

### Where to Go.

THE DOWDESWELL GALLERIES.—An exhibition of Gaspard Latoix's paintings, entitled "English Pastorals," will commence on Monday, November 20th, at the Dowdeswell Galleries.

### To Nurses.

**IN order to increase and vary the interest in the *Mirror*, we invite contributions from any of our readers in the form of either an article, a paragraph, or information, and will pay a minimum of 5s. for each contribution. All rejected manuscripts are returned in due course, and all payments for manuscripts used are made at the beginning of each quarter i.e., January 1st, April 1st, July 1st, and October 1st.**

### Winter Cloaks at Garrould's.

FEW are the establishments which a nurse can visit with greater profit and pleasure than that of Messrs. Garrould. Its attractions are as varied as they are complete, and comprise everything that the heart can desire—from professional literature downwards. In the catalogue we find all the newest works on nursing and midwifery, and on the shelves all the most up-to-date appliances in the way of bags, wallets, and surgical instruments. So comprehensive an assortment cannot fail to attract customers from all parts, and we are assured that none will go away disappointed. In lingerie there is nothing very fresh; indeed, it would appear difficult to improve upon that already in stock, but we found some new models in cloaks which struck us as eminently useful and attractive. Chief among these is the improved "St. Alban," which is cut in to the figure sufficiently to define it without being close fitting or, on the other hand, unduly baggy. The sleeves are shaped after the manner of a cape, which gives warmth as well as a graceful appearance to the wearer, and is decidedly becoming. Then there is the "Rosemary," likewise a neat and useful garment, and one that is certain to be popular with district nurses, as it is made with a deep cape, thus affording complete freedom to the arms. The "Cecil Coat" is an improvement on the cycling cloak, and certainly appears more comfortable and workmanlike than the latter. There are some charming designs in washing materials, and the colours are warranted fast. The "Milo Gingham" is a stout serviceable fabric that would stand any amount of rough wear. It is made in different coloured stripes, and also in checks. The blue and the pink stripes are especially pretty. The "Killaloe Linen" and a somewhat similar one, the "Limerick," are both useful lines, and are sure to become popular, as the shades are soft and artistic, and will wash well. Zephyr is always a charming material, but has the disadvantage of being just a little light for hard wear; this drawback has been remedied by Messrs. Garrould in their "Melville Double Warp" zephyr, which while retaining all the soft beauty of the zephyr, is considerably thicker. This advantage will be appreciated by nurses. The "Tikord" linen for aprons still retains its popularity, as it is soft, durable, and everlasting wear. Before passing on to the instrument and book department, our readers may like to be introduced to the tea-room, which is just through the mantle department, a most convenient situation, where nurses can invite their friends to visit them and enjoy a pleasant hour over the tea-cups. There is no need to hurry, as it is the wish of Messrs. Garrould's to provide for the comfort of their nurse-customers by affording them facilities for meeting and entertaining their friends. The convenience of this arrangement to those nurses who are private-nursing and have no means of seeing their relations except through such a medium, cannot be over-estimated. The room is tastefully decorated, and very comfortable. In the instrument department are many novelties, of which we can only describe a few of the most important. In wallets, which we are glad to see have nearly entirely superseded the noisy chatelaine, there are two designs that deserve especial mention—the "Eclipse" is one, and the "Eva" is the other. The "Eclipse" is aseptic, and so constructed as to rigidly exclude all dust. Its price complete is 5s. 6d., and this includes scissors, thermometer, and forceps. The district nurse's bag is wonderful value for the money, containing as it does every sort of requisite. The "Cleero" thermometer is an ingenious invention, with a violet index, and possessing the advantage of a raised tube and bulb, thus enabling it to be read without any difficulty from any part of the room. Its price is only a shilling, the usual price of these articles. There is also an excellent chart board, and a very comprehensive case-book, from the design of a nurse, on whom it reflects much credit; it is so well got up that it deserves a large sale. In conclusion, we should like to call the attention of our readers to the really beautiful little watches, with minute and second hand, the price of which commences at 25s., and which are guaranteed for three years. The red Geneva Cross on the back differentiates them from the ordinary watch and adds considerably to their attractiveness.

### Minor Appointments.

ROYAL VICTORIA HOSPITAL, NETLEY.—Miss K. A. MacManaway has been called up for army nursing duty. She was trained at the Infirmary, Crumpsall, near Manchester, and afterwards joined the Nursing Home, York Place, Manchester, for twelve months. She was subsequently connected with the Institute for Trained Nurses, High Street, and during the last six months has nursed private cases in Blackpool.

GLASGOW WESTERN INFIRMARY.—Miss Elizabeth Cochrane Sharron has been appointed Superintendent of Night Nurses. She was trained at the Glasgow Western Infirmary, and has since been charge nurse one year, and assistant matron four years, at Stanley Hospital, Liverpool; and matron for six years of Grantham Hospital.

SWANSEA GENERAL AND EYE HOSPITAL.—Miss Bessie Butler has been appointed Night Superintendent. She was trained at the General Hospital, Bristol, and has, during a period of nine years, held successively the posts of nurse, sister of female medical wards, and sister of out-patients' and casualty departments, in that institution.

CHELSEA HOSPITAL FOR WOMEN.—Miss Emma Wells has been appointed Charge Nurse. She was trained at the Norfolk and Norwich Hospital, and has since been charge nurse of the isolation block for one year, and charge nurse in the theatre and women's surgical ward in the same institution.

HOLBORN UNION WORKHOUSE INFIRMARY, CITY ROAD.—Miss M. A. White and Miss E. Roberts have been appointed Staff Nurses. Miss White was trained at Shoreditch Infirmary, and Miss Roberts at Liverpool Hospital. Both nurses have been doing private work for some little time.

GREEK HOSPITAL, ALEXANDRIA.—Miss M. Evans has been appointed Sister. She was trained at the Royal Infirmary, Sheffield, and has since been charge nurse at Fountain Hospital, Lower Tooting, London.

### TRAVEL NOTES AND QUERIES.

RULES IN REGARD TO CORRESPONDENCE FOR THIS SECTION.—All questioners must use a pseudonym for publication, but the communication must also bear the writer's own name and address as well, which will be regarded as confidential. All such communications to be addressed "Travel Editor, 'Nursing Mirror,' 28, Southampton Street, Strand." No charge will be made for inserting and answering questions in the inquiry column, and all will be answered in rotation as space permits. If an answer by letter is required, a stamped and addressed envelope must be enclosed, together with 2s. 6d., which fee will be devoted to the objects of the "Hospital Convalescent Fund." Any inquiries reaching the office after Monday cannot be answered in "The Mirror" of the current week.

THROUGH TO THE RIVIERA (Invalid).—There is a first-class carriage which goes straight through to Ventimiglia attached at Calais, so that no change is necessary in Paris. If it is necessary to go second-class you can still manage with little difficulty. By taking a ticket for the ceinture railway you need not cross Paris in a fiacre, but can reach the Lyons Station from the Nord Station with no trouble at all. (2) The train de luxe is composed exclusively of lits-salons between Paris and the Riviera, and takes first-class passengers only. A supplementary charge is made for lits-salons; from Paris to Nice £3 2s. 7d. extra, and other stations according to distance.

ROME FOR SIX MONTHS (Fiesole).—You do wisely; it is not too long to visit the Eternal City. First-class, viâ Calais, £10 13s. 7d.; second, £7 9s. 10d. A cheaper route is viâ Dieppe, first-class, £9 0s. 4d.; second, £6 6s. 2d. Clothing should be warm, much as in England, with thinner woollen suits for warmer days. Rome is brilliantly sunny, but often very cold. There is no danger of fever with ordinary care; avoid the sunset hour, and do not over fatigue yourself and go too long without food in the ardour of sight-seeing. Another danger is that of entering cold churches when heated; always carry a light wrap to put on when entering churches, which strike cold as vaults very often.

PAU (Puzzled).—There is a very considerable difference in the climate of Pau or in that of St. Jean-de-Luz and Biarritz. Pau is mild, warm, and remarkably sedative; the two latter are exhilarating and bracing. You must consult your doctor, and abide by his decision.

OBER-AMMERGAU (Sylvia).—Several answers on this subject have been given in the last few weeks, which, if you refer to them, will give you the information you require. Before long I shall be in possession of further information with regard to tickets, journey, &c. An article will also shortly appear dealing with the Passion Play.

### Notes and Queries.

**The contents of the Editor's Letter-box have now reached such unwieldy proportions that it has become necessary to establish a hard and fast rule regarding Answers to Correspondents. In future, all questions requiring replies will continue to be answered in this column without any fee. If an answer is required by letter, a fee of half-a-crown must be enclosed with the note containing the enquiry. We are always pleased to help our numerous correspondents to the fullest extent, and we can trust them to sympathise in the overwhelming amount of writing which makes the new rules a necessity.**

**Every communication must be accompanied by the writer's name and address, otherwise it will receive no attention.**

*South Africa.*

(79) Will you kindly tell me if nurses of good training can offer their services for the war in South Africa? If so, where am I to apply?—*Constance W.*

Nurses of three years' training can apply to be put on the Army Nursing Reserve. Address the Hon. Secretary, Army Nursing Reserve, 18, Victoria Street, S.W.

(80) I should be much obliged if you will let me know how I can offer myself as a nurse for the present war. I am 39, and was trained at St. Bartholomew's, London, and have been nursing eight years.—*Nurse Lucy.*

See reply to "E. R. I."

(81) Would there be any use a trained nurse offering her services in nursing the wounded in the Transvaal, and to whom should she apply?—*E. R. I.*

Apply to the Hon. Secretary, the Army Nursing Reserve, 18, Victoria Street, S.W. There is very little chance of being sent to Africa, even in the event of being accepted for the reserve.

(82) I am engaged to nurse a patient in East London (Africa) in April. Would you kindly tell me if by going earlier and by being willing to accept any Government work in the meantime, there would be any chance of getting help for my passage out? I am a fully certificated nurse, but have had no experience in army nursing; or can you tell me where I could get information on this subject?—*Stella.*

See reply to "Constance W." There is a superabundance of applications to nurse the wounded.

(83) "Nurse O'D." begs to offer her services during the war in South Africa. She has had six months' hospital training.

See reply to "Constance W."

(84) Would you kindly tell me the conditions under which nurses are admitted to the Army Nursing Service, and to whom I should apply for a form of application?—*Margot.*

See reply to "Constance W."

*Epilepsy.*

(85) I shall be glad if you will kindly state the names and addresses of two or three homes for children suffering from epilepsy? I cannot see a copy of Burdett's "Hospitals and Charities" to find out for myself.—*Surgeon.*

The West-end Hospital for Diseases of the Nervous System, Paralysis, and Epilepsy, 73, Welbeck Street, W. (especially for the treatment of children); the Home for Epileptics, Maghull, near Liverpool (office, 5, Dale Street, Liverpool); and the National Society for the Employment of Epileptics, Chalfont St. Peters (office, 12, Buckingham Street, Strand). The two latter are for the benefit of adults as well as children. At Maghull the charges are from 7s. 6d. weekly.

*Reading Association.*

(86) I should be glad if you could tell me of a reading association for nurses in which they can ask questions, or of any competent individual who would undertake to coach by correspondence theoretic, as well as practical, nursing?—*W. H. A. T.*

There is the new association formed by Miss L. G. Moberly, 24, Portland Place, W. Miss Lamport, 52, St. John's Wood Road, N.W., could advise you about coaching by correspondence.

*Refugee.*

(87) Would you tell us if it would be possible for an Armenian refugee from L'Ecole Centrale, Constantinople, with a knowledge of dispensing, to obtain a situation in England as assistant dispenser either to a doctor or in a hospital? He can speak but little English. It is an urgent case of great need, and it would be a true kindness if you would give any practical suggestion as to whom to apply. Is it committee or house surgeon who usually engages the dispenser in hospitals? *Nurse.*

We know no better way than by advertising or answering advertisements. The appointment is generally made by the committee. You should first find out if his qualification as a dispenser is recognised either by the Pharmaceutical Society, 17, Bloomsbury Square, W.C., or by the Apothecaries' Hall, Blackfriars. Vacancies in hospitals are usually advertised in medical journals.

*Massage.*

(88) Will you kindly tell me which is the best school of massage, and if one can learn the art in less than six weeks, as I want to go to London expressly for this purpose? I am a private nurse and fear I could not get leave longer than three or four weeks. Can you give me an idea of the cost of same.—*Gyp.*

Write for advice to the Secretary of the Society of Trained Masseuses, 12, Buckingham Street, Strand, W.C.

*Text Books.*

(89) Will you kindly inform me what are the best text books to read up for the London Obstetrical Examination?—*Nurse Rose.*

A "Practical Handbook of Midwifery," by Haultain (Scientific Press, price 6s.), or "Herman's First Lines in Midwifery," are highly recommended for the purpose.

*Monthly Training.*

(90) I have had particulars of the training of monthly nurses from three institutions, viz., the British Lying-in Hospital, the Clapham Maternity Home, and the City of London Lying-in Hospital. Will you kindly advise me as to which of these it would be best for a nurse (general) to train in?—*Beta.*

The training at all the schools you name is good.

*Share.*

(91) I am a midwife (L.O.S.) working alone, and wish to have another to live with me and share the work. What would be fair remuneration to offer in addition to board, lodging, and washing? I mean, what share of the profits ought she to have?—*Ignoramus.*

The share of the profits must be decided entirely by the amount of work, and the fees paid for it. Do you not think it would be best to engage an assistant nurse at a fixed salary, inserting a clause in your agreement (which you should have in writing) that she must not set up for herself in your neighbourhood? Then, after seeing how she worked, and how her work affected your income, you would be in a position to judge what would be a fair sum to offer her, or another lady, in the event of your wishing to make a new arrangement.

*Measurements.*

(92) I shall be glad to know what book would teach me the measurement of medicines.—*Mrs. F.*

You will find the quantities of each drug prescribed for the usual dose in a "Materia Medica" (Mitchell's for instance). But if you wish to know how to dispense drugs you must learn by practical work under a dispenser.

*Indian Nursing Service.*

(93) I shall be glad if you can let me know who is the proper person to whom I should write for all information about the Indian Nursing Service, length of training needed, &c.—*Mundis.*

Apply to the Under Secretary at the India Office for forms of application. Applicants must be ladies of three years' hospital training, between 25 and 35 years of age.

*Lecturer's Remuneration.*

(94) Could you tell me what I ought to charge for giving demonstrations and lectures on nursing?—*Ireland.*

A very usual charge is a guinea for a single lecture, with a substantial reduction on a course. The locality and the circumstances of the case of course naturally influence the prices.

*Male Nurse.*

(95) I should be much obliged if you could inform me as to where probationers are received and trained as male nurses (physical). Also for any further particulars which would enable me to apply with view to entering the profession.—*H. W.*

The National Hospital for the Paralysed and Epileptic, Queen's Square, Bloomsbury, W.C., is the only hospital which trains male nurses.

*Typhoid.*

(96) I shall be glad to know how many cases of typhoid were nursed by one nurse during the typhoid epidemic at Maidstone, and what was done for each patient?—*District Nurse.*

Can any of our readers kindly reply from personal experience?

*Burns.*

(97) Would you or one of your numerous correspondents kindly tell me the treatment of burns with ung. picric acid and picric acid solution? Some say the dressing should be changed every day; others say every third day. Also, does the absorption of this acid cause poisoning? As I find the patient's urine darkens often within forty-eight hours after the manner of carbol urea or carbolic poisoning through absorption.—*Perplexed.*

If the picric acid remains wet there may in some cases be a certain amount of absorption, but the ideal plan is to use it as a dry dressing. The solution of picric acid is to be made as follows: A drachm and a-half of picric acid is to be dissolved in three ounces of alcohol, which is then diluted with two pints of distilled water. The acid should be kept ready dissolved in alcohol in the strength above mentioned, and diluted with the water as required. After the burn has been cleansed as far as possible with bits of absorbent cotton soaked in the lotion, and after the blisters have been pricked with as little disturbance of the epithelium as possible, strips of sterilized gauze soaked in the above watery solution of picric acid are applied so as to cover the whole of the injured surface. A thin layer of absorbent cotton wool is then applied, and all is held in place by a light bandage. This dressing may be left on for three or four days. The difficulty is to get it off. For this purpose it must be well moistened with the picric acid solution, or better still with sterilised water. The place is then to be dressed again in just the same way, and this dressing may be left on for a week. Some surgeons use lint soaked in picric acid solution, and cover this with jaconet or some other form of waterproof tissue, and after the dressing has been removed at the end of three days, renew it daily. Others again use an ointment. There is no doubt that by so doing the removal of the dressing is rendered much easier, but the manifold advantages of a dry dressing are lost, and so far as there may be any risk of absorption, which, however, we do not think is great, it would no doubt be increased. Our experience would point in the direction of the best plan being to use the picric acid without any waterproof on the first application, so that by evaporation it may become a dry dressing, and then when it is removed at the end of the third or fourth day to be prepared to act according to circumstances. If there is but little discharge repeat the dressing, but if the discharge threatens to be profuse use a moist dressing with a waterproof covering, and renew it frequently.

*Nurses' Co-operation.*

(98) I am anxious to start private nursing on this system in a large provincial town. Will you please tell me the best way to set to work?—*Queen's Nurse.*

Write to the Nurses' Co-operation, New Cavendish Street.

